# Studies on substantially increased proteins in follicular fluid of bovine ovarian follicular cysts using 2-D PAGE and MALDI-TOF MS

**DOI:** 10.1186/1477-7827-3-23

**Published:** 2005-06-08

**Authors:** Jiro Maniwa, Shunsuke Izumi, Naoki Isobe, Takato Terada

**Affiliations:** 1 Graduate School of Biosphere Science, Hiroshima University, Higashi-Hiroshima, Hiroshima 739-8528, Japan; 2 Preclinical Sciences Department, AstraZeneca KK, Osaka 531-0076, Japan; 3 Graduate School of Science, Hiroshima University, Higashi-Hiroshima, Hiroshima 739-8526, Japan

## Abstract

**Background:**

The objective of this study was to identify substantially increased proteins in bovine cystic follicular fluid (FF) in order to clarify the pathology and etiology of bovine ovarian follicular cysts (BOFC).

**Methods:**

Proteins in normal and cystic FF samples were subjected to two-dimensional polyacrylamide gel electrophoresis (2-D PAGE) and were compared using silver stained gel images with PDQuest image analysis software. Peptides from these increased spots were analyzed by matrix assisted laser desorption/ionization-time of flight mass spectrometry (MALDI-TOF MS), and were identified based on the NCBI database by a peptide mass fingerprinting method.

**Results:**

Comparative proteomic analysis showed 8 increased protein spots present in cystic FF. MS analysis and database searching revealed that the increased proteins in cystic FF were bovine mitochondrial f1-atpase (BMFA), erythroid associated factor (EAF), methionine synthase (MeS), VEGF-receptor, glyceraldehydes 3-phosphate dehydrogenase (GAPDH), heat shock protein 70 (HSP70), β-lactoglobulin (BLG) and succinate dehydrogenase Ip subunit (SD).

**Conclusion:**

Our results suggest that these proteins are overexpressed in BOFC, and that they may play important roles in the pathogenesis of BOFC. Furthermore, these proteins in the FF could be useful biomarkers for BOFC.

## Background

BOFC is one of the most frequently diagnosed gynecological findings in cattle, and is a major reproductive problem in cows, causing infertility [1-3]. BOFC is generally defined as follicle-like structures of greater than 25 mm in diameter without a corpus luteum in both ovaries [1,4]. There have been several different hypotheses regarding the cause of BOFC, such as inherited factors, high lactation, aging, seasonal effects, nutritional condition, environmental estrogen and stress [1,2,4,5]. The primary cause of BOFC development has not been elucidated because of the variety of histological characteristics [1], various abnormal hormonal patterns [1,4], and differing therapeutic responses [1,4]. However, it is generally believed that cysts are formed in response to an "endocrine imbalance" [3,4,6]. Several hypotheses have been proposed suggesting that gonadotropin releasing hormone (GnRH) and gonadotropin are implicated in the pathogenesis of ovarian cysts. Actually, some authors attribute the disease to alterations in synthesis and release of GnRH [6,7], luteinizing hormone (LH) [7-9] or even to a receptor deficiency at the pituitary level [10,11].

2-D PAGE, originally described by O'Farrell [12] in 1975, is the method of choice for the separation of cell proteins, where proteins are separated in two sequential steps. This technique is an important proteomics tool, using which many protein spots can be visualized, resulting in a global view of a proteome's state [13]. Furthermore, recent developments in 2-D PAGE and mass spectrometry technologies have enabled quantitative analysis of differential proteomics, such as a comparison between normal and disease status, allowing identification of protein markers to characterize a specific physiological or pathological cell or tissue state [14,15] which have been used in the fields of diagnosis and biomarker identification of animal and human diseases [16,17].

As described above, research on BOFC has focused on endocrinological conditions [6,8], but there are few research reports on proteins in the FF of BOFC. Although Mortarino et al. [18] published one short research report about making a 2-D PAGE map of proteins expressed in the FF of BOFC, they used a 2-D PAGE process using FF which had not been depleted of abundant proteins such as albumin or immunoglobulin G. Therefore, it was thought that they overlooked spots of minor, but important proteins. Muranaka et al. [19] also examined protein in FF; the content of total protein in the FF of BOFC was significantly lower than that of the FF of normal follicles.

This study was designed to determine any substantially increased proteins in the FF from BOFC using a differential proteomics technique. The results help to clarify the pathology and etiology of BOCF and will contribute to the discovery of BOFC biomarkers.

## Methods

### Experimental design

This study consisted of the two following experimental phases; 1) examination of protein sample preparations from FF, and 2) assessment of increased proteins in the FF of BOFC by 2-D PAGE.

### Experiment 1: Examination protein sample preparation

Cystic follicles were collected from dairy cows at a local slaughterhouse. FF was aspirated carefully with a 20 mL syringe, and centrifuged at 10,000 × g at 4°C for 30 minutes to eliminate cells and other insoluble materials, and stored at -30°C until processing for protein sample preparation.

Three types of FF protein sample were prepared in order to find an appropriate sample preparation method for 2-D PAGE.

Type A: Non-treatment

Type B: Deplete impurities (salts, lipids, detergent or nucleic acid)

Type C: Deplete both abundant proteins (albumin and IgG) and impurities

A detailed sample preparation method for depletion of both abundant proteins and impurities for Type C is shown in the following section on "Sample preparation". Non-depleted FF was used as Type A. Deplete impurities from FF were used as Type B. Samples depleted of both abundant proteins (albumin and IgG) and impurities were used as Type C. These samples were subjected to 2-D PAGE and silver stain gel images were compared visually.

Details of sample preparation, 2-D PAGE and silver staining are shown in the latter parts of this section.

### Experiment 2: Assessment of increased proteins in the FF of BOFC

Ovaries with (n = 4) or without (n = 3) cystic follicles were used in this experiment. A follicular cyst was diagnosed when the follicle was greater than 25 mm in diameter in the absence of a functional corpus luteum in both the right and left ovaries [1]. Follicles of about 10 mm in diameter from ovaries without cystic follicles were used as large normal follicles. About 300 – 1000 μL of FF was aspirated carefully from each follicle with a syringe. After measureming progesterone and estradiol-17β in FF, FFs from cystic follicles were stored individually and those from normal follicles were pooled.

Each follicle was fixed in formalin after aspiration of FF, processed for histological examination and stained with hematoxylin and eosin using a standard method.

The FF protein samples were prepared by the Type C method (depleted of both abundant proteins (albumin and IgG) and impurities). Each sample from cystic follicles with pooled control samples was simultaneously processed for 2-D PAGE twice and gels were visualized by silver staining. The stained gels were scanned using a desktop scanner and scanned images from cystic and normal FFs were analyzed with PDQuest software version 7.1 (Bio-Rad Laboratories Inc., Hercules, USA). Expressions of protein spots on the images from cystic and normal FFs that were processed for 2-D PAGE simultaneously were compared, and a overall comparison of the results from 4 cystic follicle images was made.

Eight protein spots which were increased on cystic FF were excised from the silver stained gels and subjected to mass spectrometry analysis and protein identification.

Details of each experimental process are given in the following section.

### Enzyme immunoassay of progesterone and estradiol-17β in follicular fluid

The enzyme immunoassay procedure for progesterone and estradiol-17β was followed as described previously [20]. FF diluted with H_2_O was extracted with petroleum ether for progesterone or diethylether for estradiol-17β. The decanted ether phase was dried in a glass tube and a buffer (0.05 mol/L boric acid, 0.2% BSA) was added. These reconstituted samples were placed into wells of a microtiterplate that was coated previously with an anti-rabbit IgG antibody (ICN, Pharmaceuticals Inc., USA) followed by the addition of an anti-progesterone antibody and Horseradish peroxidase (HRP) conjugated progesterone for progesterone, and an anti-estradiol-17β antibody (Kambegawa Institute, Tokyo, Japan) and HRP conjugated estradiol-17β (Kambegawa Inst.) for estradiol-17. After 2-h incubation plates were washed and a 3,3',5,5'-Tetramethylbenzidine solution was applied to the substrate followed by reading optical density measurement at a wavelength of 450 nm.

### Protein sample preparation

Initially, the total protein content in FF was determined using a commercial protein assay kit (DC Protein Assay, Bio-Rad laboratories Inc., Hercules, USA). After measuring the protein content, high abundant proteins, albumin and immunoglobulin G (IgG) were removed from FF using an Aurum serum protein mini kit (Bio-Rad laboratories, Inc., Hercules, USA) to enable the visualization of low abundant proteins. Then, proteins in depleted FF samples were concentrated by centrifugation using a 3 kDa cut off cellulose membrane filter unit (Microcon YM-3, Millipore, Bedford, USA). Finally, impurities such as salts, lipids, detergent or nucleic acid were removed from concentrated samples using a 2-D Clean-Up Kit (Amersham Biosciences, San Francisco, USA).

### 2-D PAGE

Fifty μg of protein sample was dissolved in a sample buffer (8 M urea, 0.5% ampholine ph3.5-10, 0.5% Triton X-100, 10 mM dithiothreitol (DTT) and Orange G). The sample buffer, including 50 μg protein, was absorbed into an immobilized pH gradient (IPG) strip (Immobiline Dry Strip, 11 cm, pH range 3–10, Amersham Biosciences, Uppsala, Sweden) overnight. The rehydrated strip was subjected to isoelectric focusing on a MultiPhor II electrophoresis chamber (Amersham Biosciences, Uppsala, Sweden) for a total of 22,651 Vh at 15°C. The focused IPG strip was equilibrated in sodium dodecyl sulphate (SDS) buffer (30% glycerol, 1.0% SDS and 6 M urea in 50 mM Tris-HCl). The first equilibration step was carried out in SDS equilibration buffer with 16 nM DTT, and the second portion of the SDS equilibration buffer contained 240 mM iodoacetamide and bromophenol blue. Both equilibration steps lasted 15 min at room temperature. The equilibrated IPG strip was placed onto an 8–18% gradient polyacrylamide gradient gel (ExcelGel SDS, Amersham Biosciences, Uppsala, Sweden) for second dimensional SDS-PAGE. The SDS-PAGE was performed at 20 mA for 30 min, 50 mA for 5 min and 50 mA for 70 min at 15°C with power supply limitation of 600 V and 30 W using a MultiPhor II electrophoresis chamber. After separation in SDS-PAGE, the proteins on the gel were fixed and visualized by silver staining using a Silver Stain Plus Kit (Bio-Rad laboratories Inc., Hercules, USA).

### Mass spectrometry analysis and protein identification

The spot was excised from the silver stained gel and then digested with trypsin using a previously described method [21]. For in-gel digestion, the pieces of gel were immersed in 50% acetonitrile solution for 10 min and this process was repeated several times. After washing with acetonitrile, acetonitrile was removed and the gel pieces were dried in a vacuum centrifuge for 10 min. The gel pieces were shaken with 100 μL of reducing solution (10 mM DTT and 25 mM ammonium bicarbonate) for 60 min at 56°C. Subsequently, the gel pieces were shaken under dark conditions with 100μL of an alkylating solution (55 mM iodoacetamide and 25 mM ammonium bicarbonate) for 45 min. The gel pieces were shaken with 50% acetonitrile and 25 mM ammonium bicarbonate for 10 min twice. The solvent was removed, and the gel pieces were dried again. The gel pieces were then immersed in a digestion solution containing 12.5 μg/mL sequencing grade modified trypsin and 25 mM ammonium bicarbonate and incubated on ice for 10 min. After removing unabsorbed solution, the gel pieces were incubated for 16 h at 37°C. Digested peptides were extracted twice from gel pieces with 50 μL of 50% acetonitrile and 0.1% trifluoroacetic acid. The pooled supernatants were dried in a Speed Vac, and the peptides were dissolved in 10 uL of 50% acetonitrile with0.1% trifluoroacetic acid. MALDI-TOF MS was performed on an Ultraflex time-of-flight instrument (Bruker Daltonics, Billerica, USA) equipped with a nitrogen laser operating at 337 nm. All MALDI-TOF results were obtained in the reflector-positive mode using α-cyano-4-hydroxycinnamic acid (saturated solution in 50 % acetonitrile with 0.1% trifluoroacetic acid) as the matrix. Analytes were prepared by mixing 0.5 μL of peptide sample with 0.5 μL of matrix soln. on a MALDI plate and allowed to air dry at room temperature in a hood before inserting then into the spectrometer. Mass spectra were calibrated with angiotensin II (1046.54 Da), angiotensin I (1296.68 Da), substance P (1347.74 Da), bombesin (1619.82 Da), adrenocorticotropic hormone (ACTH18-39) (2465.20 Da), and insulin (5733.54 Da). All masses are reported as monoisotopic mass values. In addition, peptides derived from trypsin (843.02 and 2212.44 Da) were used for validation when clearly observed. Peptides were identified with the Mascot search program (, Matrix Science, London, UK) against the NCBI database to perform theoretical trypsin digests and search for potentially unmodified tryptic peptides (with up to one missed cleavage) or suspected modified species. Methionine residues were considered as either normal Met or their oxidized form (Met-ox), and cysteine residues were considered to be carbamidomethylated (C-cam) or reacted with acrylamide to produce Cys-S-b-propionamide (C-pam).

### Statistical analysis

Differences in the concentration of estradiol-17β and progesterone in follicular fluid, and the ratio of those hormone concentrations between normal and cystic follicles were analyzed by analysis of variance, followed by student's t-test. Differences were considered significant at P < 0.05.

## Results

### Examination of protein sample preparation

**Figure 1:**
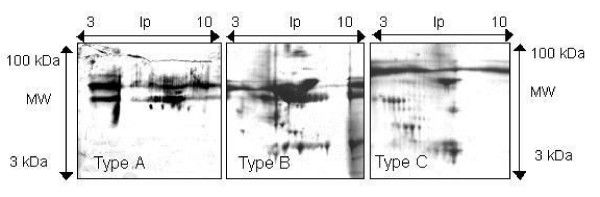
Silver stained 2-D PAGE images of proteins from bovine ovarian follicular fluid. Sample type A; Non treatment, Sample B; Depleted of impurities (salt, lipids, detergent and nuclei acid), Sample C; Depleted of abundant proteins (albumin and immunoglobulin G) and impurities.

2-D PAGE images of type A (non treatment), type B (depleted of impurities) and type C (depleted of both abundant proteins and impurities) are shown in Fig. 1. Only large streaks and some spots were seen on the gel image of type A. After removing impurities, such as salts, lipids and nuclei acid, the large streaks disappeared, but there were about 15 spots, including a large albumin spot, on the gel image of type B. By removing both abundant proteins and impurities, the image was improved, and about 40 spots were visualized on the gel image of type C.

### Assessment of increased proteins in the FF of BOFC

**Figure 2:**
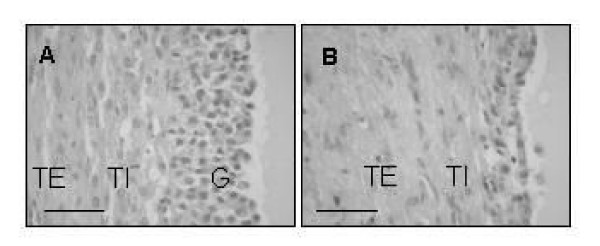
Light micrographs of bovine ovarian follicles stained with hematoxylin and eosin. [A] Normal follicle, approximately 10 mm in diameter with granulosa. [B] Cystic follicle, approximately 30 mm in diameter with theca interna, but without granulosa. G: granulosa layer, TI: theca interna, TE: theca externa. Bar = 50 μm...

On histological examination, ovarian follicles from the ovaries without cysts used as normal controls in this study (Panel A in Figure 2) were intact and were assessed to be large normal healthy follicles. On the other hand, granulosa cell layers were exfoliated and the theca internal of cystic follicles were thinner than those of normal follicles (Panel B in Figure 2), and were assessed as late stage cystic follicles.

The ratios of estradiol-17β/progesterone for three of four cystic follicles were below 1, and those of all normal follicles were above 1 (Table 1).

**Table 1:** Concentrations of estradiol-17β and progesterone, and protein content in the follicular fluid of cystic and normal follicles

	Concentration (ng/mL) of		Estradiol-17β/progesterone	Protein content (mg/mL)
			
	Estradiol-17β	Progesterone		
Normal (n = 3)	150.3 ± 115.8	57.5 ± 57.9	3.09 ± 2.85	87.6^a^
Cystic (n = 4)	59.4 ± 108.5	284.7 ± 181.8*	0.80 ± 1.55	83.1 ± 9.7

All data are expressed as mean ± standard error except for protein contents of normal follicles

^a ^Protein content was measured using a pooled sample of three normal follicles.

* Value of cystic FF is significantly different from value of normal FF (p < 0.05)

FFs from normal follicles were generally palish yellow to yellow and those from cystic follicles were dark yellow and fulvescent. This suggested interfusion of blood into the FF of cystic follicles. The total protein contents in FFs are shown in Table 1. Although the number of samples was limited, there was no particular tendency in the contents of proteins, irrespective of cysts.

**Figure 3:**
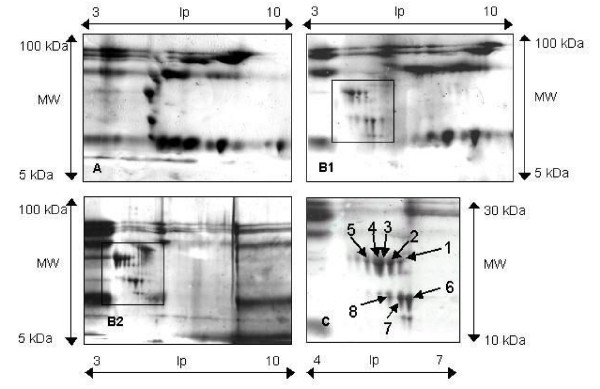
Representative silver stained 2-DPAGE images of proteins from normal follicles [A] and cystic follicles [B1 & B2]. Protein samples from both types of follicles were depleted of both abundant proteins (albumin and IgG) and impurities. Expressions of protein spots on the images from cystic and normal FFs which were processed for 2-D PAGE were simultaneously compared, and the results compared using each image image. Panel C is an enlarged image of square areas in panel B1, which contained some additional protein spots of cystic follicles.

We compared the differential expression of proteins by 2-D PAGE of FFs from cystic and normal follicles. Silver staining detected about 30–40 protein spots on images from both normal and cystic follicles (Panel A and Bs in Figure 3, respectively). There were only large spots on the image of normal follicles, while small spots of minor proteins were not detected on panel A of Figure 3. On the other hand, there were about 30 spots on the image of cystic follicles, and these included about 10 minor proteins spots on panels B1 and B2 as shown in Figure 3. Most of these spots were clumped in a square area on panels B1 and B2. An image comparison using PDQuest showed 8 increased protein spots were stably present on the all cystic FF gels (Panel C in Figure 3). All 8 spots were digested and forwarded to MALDI-TOF MS analysis for protein identification. Database searching with peptide mass fingerprinting by MALDI-TOF-MS/MS analysis identified the protein from spots No. 1 to 8 as BMFA, EAF, MeS, VEGF-receptor, GAPDH, HSP70, BLG and SD (Table 2), respectively. Although the molecular weight and isoelectric point of HSP70 calculated from the reported primary structure differed from those extrapolated by the position of the spot on the gel, it was confirmed by MS/MS amino acid sequence analysis that the protein from spot No. 6 was HSP70.

**Table 2:** List of identified proteins from the 8 spots in cystic follicles based on the NCBI database using a peptide mass fingerprinting method.

Spot^a^	Identified protein	Nominal mass (Mr)	Calculated pI value	Number of matched peptides	Sequence coverage (%)^b^
1	Bovine mitochondrial f1-atpase	13558	10.09	3	17
2	Erythroid associated factor	10711	4.82	2	26
3	Methionine synthase	14618	6.1	3	35
4	VEGF-receptor (Fragment)	17481	4.52	3	34
5	Glyceraldehydes 3-phosphate dehydrogenase (Fragment)	16813	9.21	3	33
6	Heat-Shock Cognate 70 kd Protein 44 kd Atpase N-Terminal Mutant With Cys 17 Replaced By Lys	41580.5	7.8	4	14
7	β-lactoglobulin	18156	4.84	4	36
8	Succinate dehydrogenase Ip subunit	16176	5.87	4	33

^a ^The numbers correspond to the spot number on the gel shown in Figure 4.

## Discussion

It is reported that bovine FF includes abundant protein, such as albumin and IgG [19]. Mortarino et al. [18] carried out 2-D PAGE on the FF of BOFC, which was not depleted of these abundant proteins, so they overlooked some spots of minor, but important proteins. Therefore, the effect of removing abundant protein and impurities, such as salts, lipids, detergent and nucleic acid, from FF on 2-D PAGE was examined in the present study in order to obtain satisfactory 2-D PAGE images for differential proteomics. The quality of the 2-D PAGE images was improved and the number of visualized spots increased by removing abundant proteins, such as albumin and IgG from FF, as well as other body fluids, serum [22,23], cerebrospinal fluid [24] and bile [25], and by removing impediments, such as salts, nucleic acid and lipids, for the isoelectric focusing process [26] in this study. Concomitant to the removal of albumin and IgG, there was a significant enhancement in the staining intensity of several lower abundance protein spots. This indicates that 2-D PAGE using refined FF is a useful experimental tool for research on the physiology of FF in the ovaries of farm and experimental animals.

The sample ovarian follicles used in this study were examined morphologically and endocrinologically. The diameters of cystic follicles were greater than 25 mm by macroscopic observation; the granulosa cell layers were already exfoliated and the theca internals of some cystic follicles were thinner than those of healthy follicles. These observations show that the follicles examined in this study were late stage cystic follicles [1,27-29]. Non-dominant regressing cystic follicles showed lower estradiol-17β concentrations and higher progesterone concentrations compare to normal follicles or dominant cystic follicles, so that the ratio of estradiol-17β/progesterone of non-dominant regressing cystic follicles was below 1 [30]. The ratios of estradiol-17β/progesterone of three of four cystic follicles in this study were below 1 and these values are consistent with the histological examination, i.e. that these follicles were late stage cystic follicles.

This study was designed to identify the specific proteins in the FF of BOFC in order to help clarify the pathology of BOFC using 2-D PAGE. We found 8 additional protein spots on the gel from the cystic follicles compared with the normal control, and these proteins were identified as BMFA, EAF, MeS, VEGF-receptor, GAPDH, HSP70, BLG and SD using the MOLDI-TOF MS technique.

Heat shock proteins (HSPs) are encoded by genes whose expression is substantial during stressful conditions, such as heat shock, inhibition of energy metabolism, exposure to heavy metals, oxidative stress, and inflammation [31,32]. Under these conditions, HSPs increase cell survival by protecting and disaggregating stress-labile proteins. Under no-stress conditions, HSPs have multiple housekeeping functions, such as folding and translocating newly synthesized proteins [32,33]. It was confirmed that HSP70 was clearly expressed in the granulosa cells of human ovaries in vitro [34], and was synthesized by oocytes and cumulus cells in cows [35], and by granulosa cells within follicles in rats [36]. HSPs play an important role in fertilization and early embryonic development [37-39]. Recently, the ability to inhibit apoptosis has become widely recognized as a function of HSP70, and this ability may contribute to its protective effect against cell death [39-42]. HSP70 inhibits apoptosis and this effect may be ascribed to its neutralizing interactions with several proapoptotic effectors [40]. Isobe and Yoshimura [43] reported that in the theca interna of bovine ovarian follicles, a high frequency of apoptosis was noted in the early cystic follicles, whereas this frequency decreased in late cystic follicles. They concluded that the decrease in the rate of cell apoptosis may be responsible for the delay in follicular regression, and that control of apoptosis may be essential for reducing the incidence of cystic follicles. This observation is consistent with our result that HSP70, which decreases apoptotic cell death, was increased in the FF of cystic follicles. Peter [3] emphasized that understanding the mechanisms that regulate granulosa cell growth, differentiation and apoptosis is of great clinical importance because aberrant signalling in any of these pathways is likely to be related to in cystic ovaries. HSP70 might be the key protein related to apoptosis in granulosa cells. It is thought that cows with follicular cysts are exposed to various kinds of stress such as oxidative stress [29], negative energy balance, poor liver function and low circulating insulin-like growth factor-I [44]. Although the relationship between these stresses and the increase in HSP70 in FF in this study is unclear, it is speculated that over-expression of HSP70 decreases apoptosis in the theca interna and leads to delayed regression of cystic follicles which had prevented ovulation due to hormonal imbalance.

VEGF is known as a hypoxia-inducible mitogen for endothelial cells [45], and is also referred to as a vascular permeability factor [46], affecting vasodilatation and capillary permeability and stimulating endothelial cell growth and angiogenesis in vivo. VEGF and its receptors have been found in the ovaries of numerous species including bovine, ovine, porcine and monkey ovaries, and may be important regulators of ovarian angiogenesis [47]. In humans, VEGF is implicated in the etiology of serious reproductive disorders such as polycystic ovary syndrome, and in this syndrome, elevated VEGF may interact with its receptors, such as Flk-1/KDR, in the affected ovaries, preventing granulosa cell apoptosis and the resultant follicle atresia, thus contributing to the growth and persistence of a large number of follicles [45]. Isobe et al. [48] reported that the numbers of positive vessels and areas with von Willebrand factor, an indicator of endothelial damage, were significantly reduced in the theca of follicles in association with the cyst development. This suggested that fewer degenerative changes in the vasculature occur in BOFC, which allows a consistent blood supply and may result in maintaining follicular tissue stability and continuation of FF accumulation. The increase in receptors of VEGF, which is well known as a mitogen for endothelial cells and a vascular permeability factor, in the present study may have been associated with abnormal follicular growth via a steady blood supply and accumulation of FF originating from serum in the BOFC.

BMFA is the water-soluble component of ATP synthase, and the enzyme plays a central role in energy transformation in most living organisms [49]. SD is a key enzyme involved in both the tricarboxylic acid cycle and oxidative phosphorylation as part of the mitochondrial respiratory chain [50]. MeS is an important cellular housekeeping enzyme [51] and methionine is the most limiting amino acid for growing cattle [52]. Therefore, fluctuations in amino acids or energy metabolism in cows with cystic follicles may lead to increased BMFA, SD and MeS. However, there is no specific information on the three proteins or EAF, GDP and BLG in ovarian physiology, follicular development and/or regression, and the exact relationship between cystic follicles and these proteins remains to be clarified.

The majority of proteins identified in the follicular fluid of cystic follicles in the present study were not the type of factors one would expect to find secreted into follicular fluid; they were predominantly intracellular proteins. Isobe and Yoshimura [43] reported that apoptotic sells in the granulosa layer were observed in cystic follicles, and granulosa cell layers were also exfoliated in this study. Therefore, these intracellular proteins might be passed into the FF from the degenerated granulosa layer. On the other hand, the cystic follicular fluids used in this study were dark yellow and fulvescent, suggesting interfusion of blood into the FF of cystic follicles, and EAF was increased in cystic FF. Thus, the possibility that these proteins came via blood cannot be ruled out. Further investigations regarding the source of these proteins and the mechanism by which they entered the FF are needed.

## Conclusion

In conclusion, although we mainly examined late stage cysts and it may be only one scene of complicated BOFC formation, we identified 8 increased proteins in the FF of BOFC using 2D-PAGE and the MOLDI-TOF MS technique. Our results suggest that these proteins play important roles in the etiology of BOFC, and that over- expression of HSP70 may contribute to delaying follicular regression by decreasing apoptosis in the theca interna, and over- expression of VEGF-receptors may be associated with abnormal follicular growth and accumulation of FF. Furthermore, the 8 proteins found in the FF could be used as biomarkers for ovarian cysts. Further studies are needed to clarify the pathology and etiology of BOFC including the examination of the source of these proteins, their interaction, the relationship between these proteins and ovarian physiology, and FF from various stages of follicles, including atretic follicles and dominant cysts.

## Authors' contributions

JM was responsible for the design, coordination of the study and experiments He performed hormonal measurements, 2D-PAGE, and protein identification, participated in the analysis of data and in drafting the manuscript. SI collaborated in the MOLDI-TOF MS analysis and protein identification. NI collaborated in the hormonal measurements and histological examinations. TT was responsible for design and coordination of the study. He analyzed the data and drafted the manuscript. All authors read and approved the final manuscript.
